# The Effect of Patency Files on Apical Canal Anatomy Using SEM Imaging

**DOI:** 10.1155/2023/7195512

**Published:** 2023-08-17

**Authors:** Michael S. Cavender, Christopher Waters

**Affiliations:** ^1^Department of Endodontics, School of Dentistry, West Virginia University, 1080A Health Sciences Center North, Morgantown, WV 26506, USA; ^2^Department of Dental Research, School of Dentistry, West Virginia University, 106a Health Sciences Addition, PO Box 9448, Morgantown, WV 26506, USA

## Abstract

**Introduction:**

There are many reasons to maintain apical patency during routine endodontic treatment. Thousands of canals are treated utilizing a patency file every year all around the world. The effect patency has on the apical anatomy of the root has been controversial for generations.

**Objective:**

This ex vivo descriptive study was created to show the effect patency files actually have on the apical root canal anatomy using visually detailed SEM images supported by dental radiographs.

**Materials and Methods:**

Three extracted maxillary anterior teeth that represent the multitude of canals in vivo were instrumented utilizing patency files. Two of the three maxillary anterior teeth were instrumented with hand files, the other maxillary anterior tooth with a .06 taper rotary file. The teeth were then scanned with an electron microscope to view the effect that the instruments had on the apical canal anatomy. A fourth tooth, a maxillary second molar, was shaped with an .06 taper rotary file and cone fitted. This tooth was radiographed with a gutta percha cone fitted to reveal the position of the narrowest constriction after patency was achieved.

**Results:**

The patency files, both hand files and rotary, were shown not to adversely affect the apical canal anatomy. Additionally, the SEM's revealed a precise demarcation of cementum to dentin which was at the root surface after patency was achieved.

**Conclusion:**

The patent use of greater tapered rotary files provides a clear demarcation of the CDJ which allows a precise acquisition of the narrowest constriction of the canal with the use of an electronic apex locator for establishing the ideal working length and precision placement of a gutta percha cone.

## 1. Introduction

Obtaining patency during root canal canal instrumentation has been controversial for over 50 years. By 1997, almost half of the dental schools in the USA teach the appropriate use of patency files [1]. Patency during root canal therapy is the technique of passing a small file through the apical foramen during cleaning and shaping [2]. Patency keeps the canal free of debris and maintains a glide path to the full extent of the canal. Lack of apical patency and the resultant blocked canal can have a detrimental effect during endodontic treatment. It has been shown that a blocked canal has a negative effect on the accuracy of apex locators [3]. Pulp debris left in the canal can support the growth of bacteria [4].

Apical patency in the past was suggested to cause several potential issues with root canal treatment and all files were recommended to be confined within the root canal space. However, the microorganisms responsible for infection were actually found in the most apical part of the root canal [5]. This is why removal of bacteria in the apical area is the main goal of retreatment in failed endodontic cases [6]. Apical patency actually improves the outcome and success rate of root canal treatments [7]. Patency allows the drainage of pus and inflammatory exudate which provides a favorable condition for the host defense mechanism to begin repair [8, 9]. Loss of apical patency can cause a loss in working length and transportation of the foramen in curved canals [10]. When it comes to irrigants cleaning the apex, apical patency prevents vapor lock allowing the irrigant into the apical 2 mm of the canal [11].

Several studies show that apical patency has no influence on the level of postoperative pain [12–14]. In fact, patency was actually associated with less postoperative pain in teeth with necrotic pulps and apical periodontitis than were the teeth treated with nonapical patency [15]. Shubham et al. [16] implied that apical patency does have an effect on postoperative pain; however, it was found that pupal status and preoperative pain were influencing factors. Furthermore, Abdulrab et al. [17] concluded that maintaining apical patency during routine endodontic treatment was not associated with an increase incidence of postoperative pain.

No study yet has shown the tooth apex anatomy after cleaning and shaping with a patency file. Thus, the purpose of this ex vivo descriptive study was to show the effect of patency files on the apical foramen with respect to location of the narrowest constriction and potential for external transportation of the foramen using visually detailed scanning electron microscopy (SEM) images supported by dental radiographs.

## 2. Materials and Methods

This study received West Virginia University Institutional Review Board acknowledgement as Non-Human Subjects Research (protocol number 2306791491).

### 2.1. Preparation of the Samples

Four extracted teeth were acquired from the WVU School of Dentistry Research Labs Tooth Depository to conduct the ex vivo descriptive study. Three maxillary anterior teeth (sample #1–3) and one maxillary second molar (sample #4) were selected randomly. The full extent of the clinical crown and the root were then evaluated. The maxillary anterior teeth were free of caries and restorations. The maxillary second molar had cervical decay present. The teeth were cleaned of external debris and sterilized in an autoclave.

A standard endodontic access was prepared in hand using a #1958 bur in a highspeed air driven handpiece in all samples. Air and water spray were used while utilizing a Global surgical microscope. Once the canals were located, the chamber was irrigated with tap water. All hand files used in this study (#6, 8, 10, 15, 20, 25, 30, 35, 40) were .02 tapered Flexofile® stainless steel K-files (Dentsply Maillefer, Ballaigues, Switzerland). The rotary files (#20, 45, 50) used were constant tapered .06 taper Vortex Blue® nickel–titanium rotary files (Dentsply Tulsa, Tulsa, USA).

Two of the three maxillary anterior teeth (sample #1, 2) were instrumented with hand files, the other maxillary anterior tooth (sample #3) with a .06 taper rotary file. In sample #1, a #10 hand file was introduced until the file was seen visibly protruding through the apical extent of the canal. Patency was achieved again using a #15 file, followed by a #20 while irrigating with water between each file. Each of the preceding hand files fit loosely. A #25 file fit snuggly and could be visualized at the apical foramen.

In sample #2, a #10 hand file was introduced until the file was seen visibly protruding through the apical extent of the canal. Patency was achieved again using a #15 file, followed by a #20, a #25, and a #30 while irrigating with water between each file. Each of the preceding files fit loosely. A #35 file fit snuggly and could be visualized at the apical foramen.

In sample #3, a #10 hand file was introduced until the file was seen visibly protruding through the apical extent of the canal. Patency was achieved again using a #15 file, followed by a #20, a #25, a #30, a #35, a #40, while irrigating with water between each file. Each of the preceding files fit loosely. A rotary file, #45 .06 taper Vortex Blue® was then placed in a torque-controlled electric handpiece which was set to the manufacturer's specifications. The canal was irrigated with water. The #45 .06 taper Vortex Blue® was introduced in the canal until the file was visualized patent. A #50 .06 taper Vortex Blue® file was then introduced in the canal by hand and fit snuggly at the apical foramen.

### 2.2. Ex Vivo Analysis

The three maxillary anterior teeth (sample #1–3) were sectioned horizontally at the apical third of the root. This was necessary to allow the root to fit on the scanning electron microscope mount within the SEM. The samples were sputtered with Gold–Palladium for a conductive surface. A Hitachi SEM S4700 (Hitachi, Tokyo, Japan) at 5 kV was used to image the teeth. Each of the empty root apices were scanned with the electron microscope ranging in image magnification from 12.0 mm × 40 SE(M) to 12.0 mm × 1.30 k SE(M) or 40x to 1,300x magnification.

The files (hand and rotary) that fit snuggly at the apex in each of the three maxillary teeth were placed in the canals and also sectioned to fit on the scanning electron microscope mount within the SEM. The scanning electron microscopy images were repeated as before at 40x to 1,300x magnification with the files visibly present at the apex.

A fourth tooth (sample #4) the maxillary second molar, had the chamber accessed and the mesiobuccal canal instrumented from a #6 through a #15 hand file until patent. This achieved an unobstructed glide path past the root surface. Then, a #20 .06 tapered Vortex Blue® rotary file was used until patency was confirmed by visually locating the tip of the file outside the root. This was performed while irrigating with tap water. The canal was cone fitted to the visual root surface using Ultimate Dental Microtipped Gutta Percha® point size medium (Ultimate Dental, Memphis, USA). Radiographic images were taken in the buccal to lingual and mesial to distal direction to capture and demonstrate the location of the newly generated apical foramen.

## 3. Results

### 3.1. Scanning Electron Microscopy (SEM)

The following images are the actual SEMs of three maxillary anterior teeth (sample #1–3) after the hand and rotary files were introduced in the canal.

**Figure 1:**
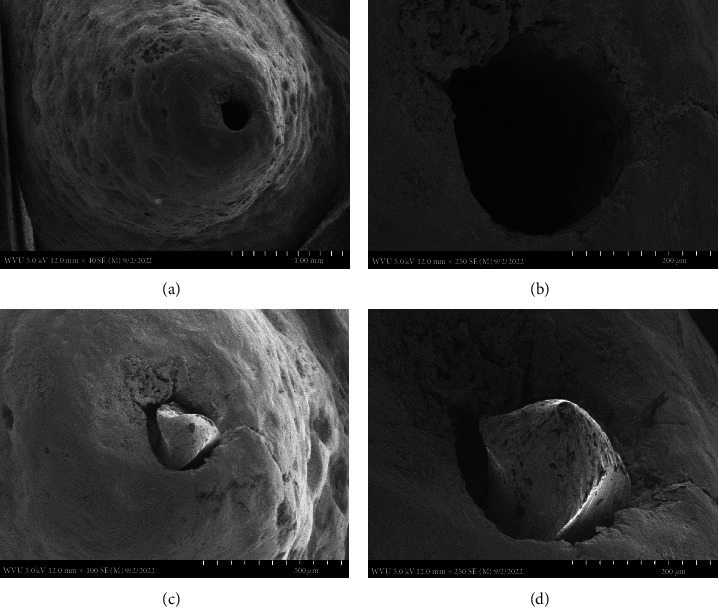
Four SEM images of the apical canal anatomy. Top SEM images (a: at 40x magnification and b: at 250x magnification) are of the canal after a #20 hand file was patent. Bottom SEM images (c: at 100x magnification and d: at 250x magnification) are of the #25 hand file fitting snuggly.

In Figure 1, sample #1 had a very small canal. The SEM revealed the apex of the canal after a #20 hand file was passed through the apex. A smooth transition from dentin to the cementum root surface was observed. The canal was not completely cleaned and there was no evidence of a minor foramen present within the canal by simply using a #20 .02 tapered hand file passively. Even with a file tip diameter of only .2 mm passing through the foramen, the canal was neither internally transported nor externally transported.

**Figure 2:**
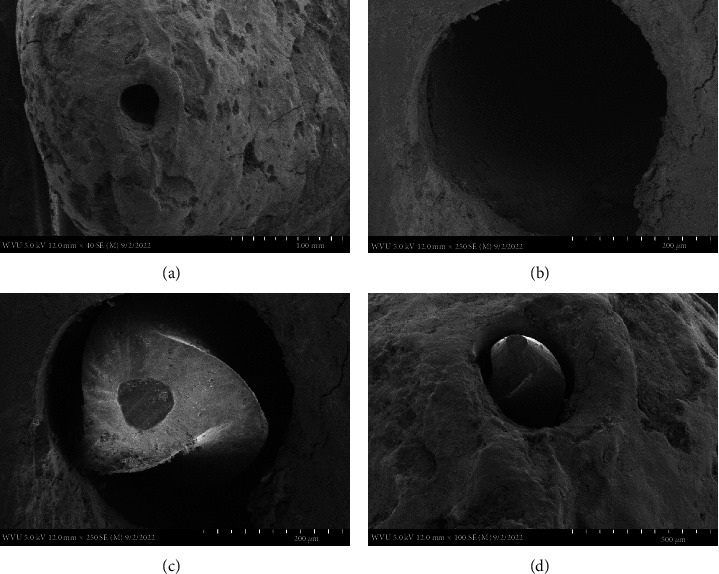
Four SEM images of the apical canal anatomy. Top SEM images (a: at 40x magnification and b: at 250x magnification) are of the canal after a #30 hand file was patent. Bottom SEM images (c: at 250x magnification and d: at 100x magnification) are of the #35 hand file fitting snuggly.

In Figure 2, sample #2, the original canal was larger than the canal in sample #1. A #30 .02 tapered hand file was used until patent. The SEM revealed a smoother transition from the dentin to the cementum root surface. Less debris was noted along the walls and a more defined, rounded canal was being created. The cementodentinal junction (CDJ) was more defined at the root surface. Similar to Figure 1, sample #1, a minor foramen was not visualized inside the canal and there was no evidence of canal transportation.

**Figure 3:**
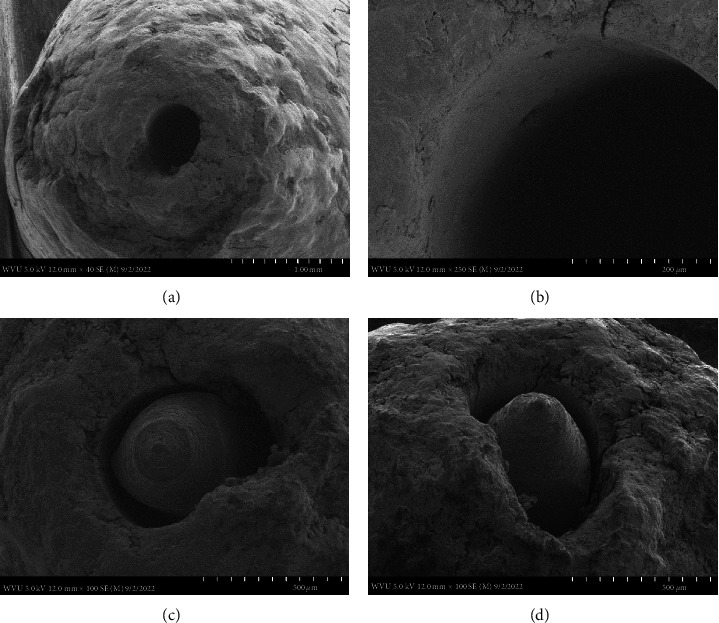
Four SEM images of the apical canal anatomy. Top SEM images (a: at 40x magnification and b: at 250x magnification) are of the canal after a #45 .06 taper rotary file was patent. Bottom SEM images (c: at 100x magnification and d: at 100x magnification) are of the #50 .06 taper rotary file fitting snuggly.

In Figure 3, sample #3, the root had the largest canal. The SEM revealed a canal that was instrumented up to a #45 .06 taper rotary file patent. The canal walls were smooth and a very distinct line angle existed between the dentin and the cementum on the root surface (B). A minor foramen was not visualized within the canal and a thin smear layer coated the walls of dentin. The canal was not transported, neither internally nor externally.

**Figure 4:**
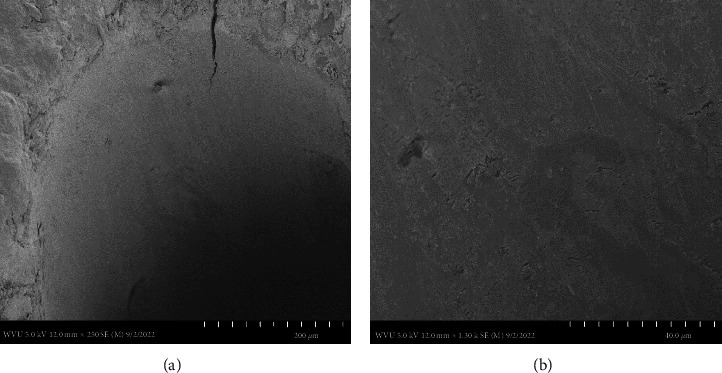
Two SEM images of the apical canal anatomy. SEM image (a) reveals a smear layer created by the #45 .06 taper rotary file at 250x magnification. SEM image (b) reveals the dentinal tubules sealed by the smear layer at 1300x magnification.

In Figure 4, sample #3, the SEM image A: showed a smear layer was created by the action of a #45 .06 taper rotary file while only irrigating with tap water. Even though the smear layer was not the aim of the study, the SEM image showed how a patent rotary file will precisely create a smear layer along the dentin wall right to the edge of the cementum covering the external root surface. The SEM image B: showed the dentinal tubules sealed by the smear layer.

### 3.2. Dental Radiography (X-Rays)

**Figure 5:**
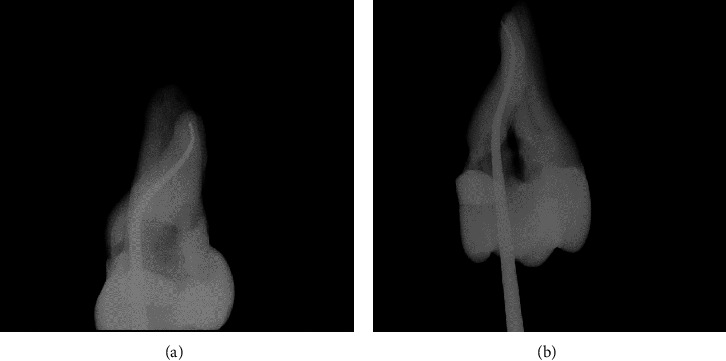
Radiographic images of an extracted maxillary second molar. (a) Gutta percha cone fitted and viewed in a buccal to lingual direction. (b) Gutta percha cone fitted in a mesial to distal view.

In Figure 5, sample #4, radiographic image A: showed an extracted maxillary second molar that had the buccal root cleaned and shaped by a #20 .06 tapered rotary file to visual patency. The canal was cone fitted in hand with a medium gutta percha cone to the visualized root surface. The fitted cone in this image appeared to be over a millimeter short of the radiographic terminus of the root. This radiographic image was in a buccal-lingual direction.

The radiographic image B: showed the extracted maxillary second molar simply rotated 90°. The medium gutta percha cone was fitted to the narrowest constriction and was viewed in a mesial–distal direction. This image demonstrated that by taking a constant tapered rotary file patent, the canal now had a constant .06 taper from the orifice to the root surface.

## 4. Discussion

In 1955, Kuttler [18] conducted a histologic anatomy study. He described the apical anatomy of the root canal system, including a major and minor apical foramen at the apex. He determined that the minor apical foramen was located at the apical constriction of the canal formed by the CDJ [18]. He recommended taking the average distance from the anatomic apex that the constriction occurred in relation to an arbitrary apical level of the root and ending the treatment at that location [18, 19]. For many years, this was the location at which the apical extent of root canal therapy was to be performed. The problem with this location is that this is a histologic location and cannot be discovered clinically [19, 20]. This method of length determination was not very precise. In fact, Wu et al. [19] stated that Kuttler's classic apical canal anatomy is more conceptual than actual as other authors have shown that the apical foramen is not located at the apex due to the variation in distance between the apical foramen and the radiographic apex.

In 1974, Schilder [21] stated in the fourth mechanical objective of cleaning and shaping that the apical foramen should remain in its original spatial relationship both to bone and to the root surface. He believed that cleaning and shaping to the radiographic terminus of the canal was a more definitive way to ensure that the canal was cleaned in its entirety, accepting that some canals will be instrumented beyond the constriction. This concept of maintaining apical patency required a file to pass through the apical constriction to free the apex of debris and contaminates. He understood that if not performed carefully, the patency file can lead to canal transportation and cause incomplete cleaning and shaping; therefore, treatment failures [21]. This method of length determination was not very precise as well.

In 2002, Goldberg and Massone [22] observed the apices of roots that were previously instrumented patently with hand files utilizing photographic transparencies mounted in slides that were projected onto drawing paper secured to a countertop. This method of canal observation was not very precise and the photographic evidence was not included in the article. The SEM images shown in this study display clear and distinct information on the effect that a patent file had in the apical extent of a canal.

In 2005, Castellucci [23] reported that in 48% of cases the apical foramen was located at the actual radiographic terminus of the root. In 40.9% of cases, the apical foramen emerged from the canal mesial or distal and yet was still radiographically identifiable. However, in 11.1% of cases, the foramen emerged on the buccal or lingual surface and therefore was not radiographically visible. Since the canal anatomy is extremely variable, the most precise method for determining the canal length is by using an electronic apex locator. Dr. Castellucci labeled this method of length determination as finding the “electronic apex.”

Today, maintaining canal patency is an important part of canal cleanliness, preventing canal blockage and is required for proper use of an electronic apex locator. Because of the variation in the location of an apical foramen and the inability to discover this location in 11.1% of all cases with a radiograph alone [23], the use of an apex locator to determine the narrowest constriction of the canal is warranted on a regular basis. This belief was also held by Mounce [24], who in 2005, mentioned reliance on a single method, especially radiographs alone, could have problems, hence an electronic apex locator should be used. However, in the cases with immature root formation or resorption, an electronic apex locator will not work properly and radiographs are still the standard of care.

Currently, to our knowledge, there are no visually detailed SEM images of the tooth apex anatomy after cleaning and shaping with a patency file. Hence, four extracted teeth, three maxillary anterior and one maxillary second molar, were randomly selected to show what effect patency files actually have on the apical root canal anatomy using visually detailed images. Apical patency is an established and widely practiced preparation technique defined by the American Association of Endodontists as a way for maintaining the apical portion of the canal free of debris using a fine file through the apical foramen [16, 25]. A statistical test was not needed to show patency as the study was an ex vivo descriptive study of an established preparation technique based on methodology in scientific research using SEM. Therefore, a power analysis and sample size were not required as there were no independent variables, variables to predict dependent variables or outcomes, measurements for quantification, or relationships between endpoints to identify potential significant differences. In addition, sample size is not a critical determinant in descriptive studies such as this, unlike comparison studies or passing-bablok and deming regression studies that rely on linear regression for method comparison and sample size estimates to get a desired outcome [26].

This preparation technique does however require precise knowledge and understanding of the corresponding internal root canal morphology and if done correctly results in the same outcome with the patency file. Therefore, to maintain a well-controlled, independent evaluation of the tooth apex anatomy after cleaning and shaping with a patency file, a single dental operator (Endodontist) performed all the experimental procedures including dental radiography, while the SEM images were individually captured blind by the Electron Microscopy Facilities Manager. The SEM images used, at the magnifications presented (Figures 1–4), were effective in showing how a patency file fit snuggly and could be visualized at the apical foramen which was confirmed by visually locating the tip of the file outside the root. Due to the high reproducibility and standardization of the presented technique, datasets of a large sample size were not needed, as Figures 1–3 are able to represent the multitude of apical root canals treated in vivo no matter the sample size. This was further supported by dental radiographic images (Figure 5), taken in the buccal to lingual and mesial to distal direction to capture and demonstrate the location of the newly generated apical foramen.

This small sample size easily identifies, as well as provides, meaningful evidence on patency that is enough to make a noticeable statement for any size population as our results were successful in highlighting patency using visually detailed SEM images supported with dental radiographs. Our SEMs show that after cleaning and shaping utilizing patency files, the minor and major foramina blend to form a single foramen at the root surface. The SEM images also show a smooth transition of the canal to the root surface where the cementum and dentin merge at sharp line angles giving the canal a very machined appearance. This allows a precision acquisition of the narrowest constriction with an electronic apex locator which has been developed by the use of patency files. This has been well documented [20, 27]. These images in effect show how maintaining canal patency utilizing hand files or rotary files have a nondetrimental effect on the apical root canal anatomy.

## 5. Conclusion

The patent use of greater tapered rotary files provided a clear demarcation of the CDJ. This demarcation allows for a precise acquisition of the narrowest constriction of the canal with the use of an electronic apex locator for establishing the ideal working length and precision placement of a gutta percha cone.

## Data Availability

The data used to support the findings of this study are included within the article.
